# Mapping the Glyco-Gold Nanoparticles of Different Shapes Toxicity, Biodistribution and Sequestration in Adult Zebrafish

**DOI:** 10.1038/s41598-017-03350-3

**Published:** 2017-06-26

**Authors:** Sivakoti Sangabathuni, Raghavendra Vasudeva Murthy, Preeti Madhukar Chaudhary, Balamurugan Subramani, Suraj Toraskar, Raghavendra Kikkeri

**Affiliations:** Indian Institute of Science Education and Research, Dr. Homi Bhabha Road, Pune, 411008 India

## Abstract

Glyconanotechnology offers a broad range of applications across basic and translation research. Despite the tremendous progress in glyco-nanomaterials, there is still a huge gap between the basic research and therapeutic applications of these molecules. It has been reported that complexity and the synthetic challenges in glycans synthesis, the cost of the high order *in vivo* models and large amount of sample consumptions limited the effort to translate the glyco-nanomaterials into clinical applications. In this regards, several promising simple animal models for preliminary, quick analysis of the nanomaterials activities has been proposed. Herein, we have studied a systematic evaluation of the toxicity, biodistribution of fluorescently tagged PEG and mannose-capped gold nanoparticles (AuNPs) of three different shapes (sphere, rod, and star) in the adult zebrafish model, which could accelerate and provide preliminary results for further experiments in the higher order animal system. ICP-MS analysis and confocal images of various zebrafish organs revealed that rod-AuNPs exhibited the fast uptake. While, star-AuNPs displayed prolong sequestration, demonstrating its potential therapeutic efficacy in drug delivery.

## Introduction

Carbohydrate on the cell surfaces has been recognized as the first line of contact for pathogens, toxins and cells^1^. Nature has utilized multivalent binding between carbohydrate and proteins to increase the avidity of cell signaling, molecular recognition and inflammations. Hence, mimicking multivalent interactions using synthetic nanoparticles can lead to potential therapeutic models to treat inflammations, imaging, bacterial infections, and cancer^2–5^. Till date, the mouse model has been extensively used as *in vivo* in all these glyconanotechnology research^6–12^. However, owing to a significant amount of sample consumption, and complex biological system, there is a need for an alternative information rich, simple *in vivo* system to reduce quantity of materials consumption and at the same time acquiring useful biological information at inexpensive, before planning the experiment with the complex models. Animals including fish, insects and worms are considered to be the simple model for pre-clinical research and each of these *in vivo* tools has their own merits and demerits. For example, *Caenorhabditis elegans* is widely used to investigate the chemical toxicity due to its sensitivity to oxidative stress^13^. Moreover, they have a short lifespan, transparency, ease of cultivation, and high-level conservation of the vertebrate genome. However, the lack of specific organs (eyes and ears) and specific tissues (bones) limits its applications for further pharmacokinetics studies. Similarly, the invertebrate *Drosophila melanogaster* and Hydra have also received similar attention^14–16^. Drosophila offers various organs similar to human systems including the digestive system, blood vessels and kidney, and high genetic homology with the human genome. However, fruit flies have lack of vital organs such as kidney, liver and spleen. In contrast, zebrafish has been used as a model in developmental biology and wide range of human diseases, including cancer, cardiovascular disorders, neurological diseases, liver diseases and immunological studies^17–20^. Zebrafish is considered to be one of the simple animal models with widely accepted ethical principles and cost of each experiment is expected to be less than a dollar. Currently, zebrafish model was used to study the human diseases, including cancer, cardiovascular disorder, neurological diseases, liver diseases and immunological studies^21–23^. In addition to study the human disease, zebrafish model was also used in nanotechnology research. Li and his coworkers have used zebrafish model to evaluate the neural behavior of silica nanoparticles. Similarly, Zhu *et al*. used fish model to demonstrate the toxicity of gold nanoparticles decorated with positive, negative and neutral charged ligands. Recently, Kovriznych *et al*. have shown that zebrafish model can be used for optimize the toxicity of the different types of nanoparticles^24–27^. However, the shape dependent gold nanoparticles toxicity, biodistribution and sequestration has not studied well so far in zebrafish model. Herein, we report the potential application of adult zebrafish in glyco-nanomaterial research. As a prototype, we report how different shapes of PEG and mannose capped goldnanoparticles (AuNPs) influence the toxicity, uptake and clearance in the zebrafish model. Previously, it has been shown that shape of the glyco-gold nanoparticles significantly influences carbohydrate-mediated bacterial adhesion and endocytosis in mammalian cells^28^. Hence, deciphering the role of different shapes of gold-nanoparticles in *in vivo* system undoubtedly results in designing better glycoprobes to target or inhibit the carbohydrate-protein interactions (CPI).

## Results and Discussion

### Synthesis and characterization of Fluorescein - conjugated glyco-gold nanoparticles

**Figure 1:**
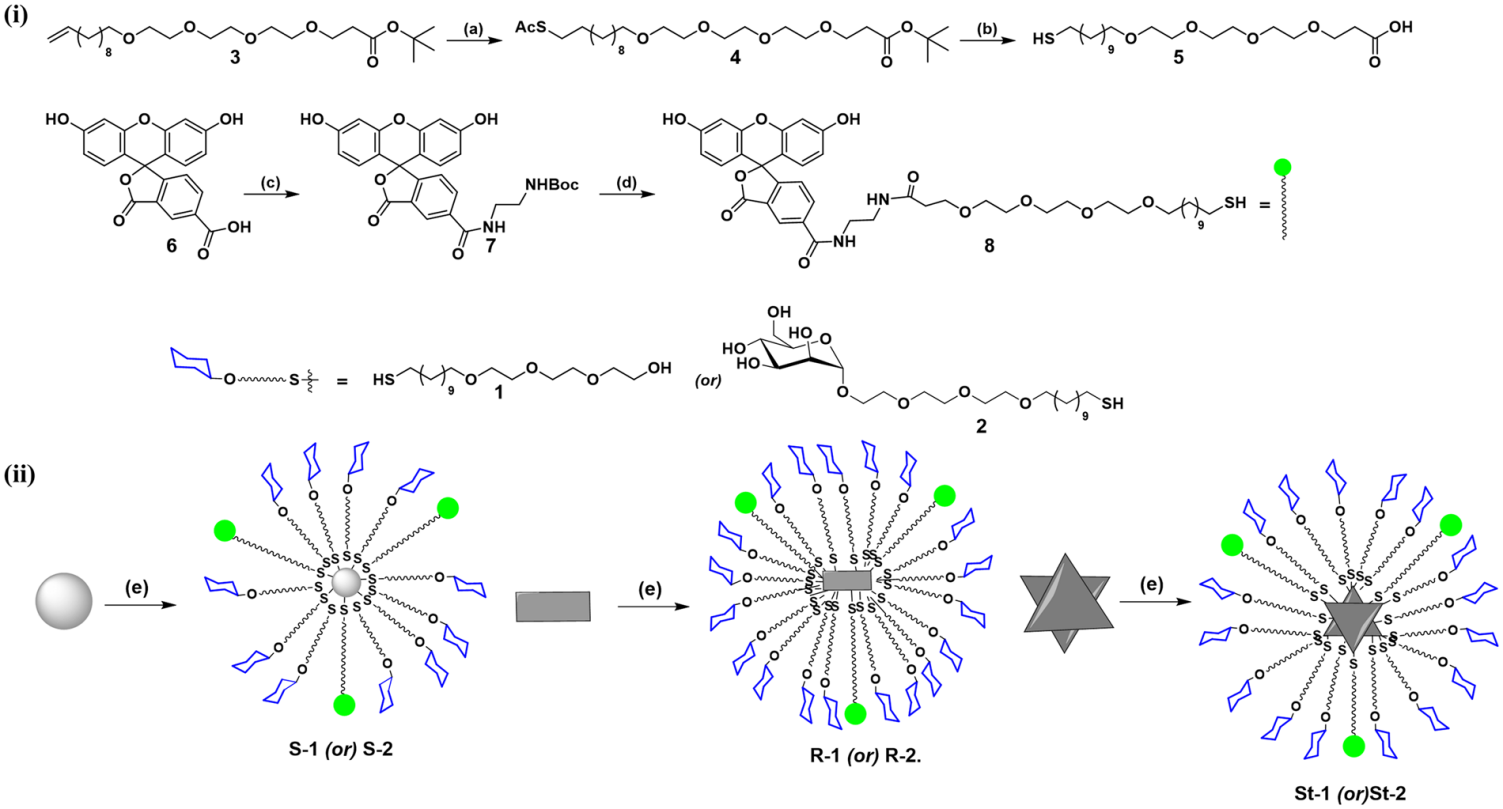
(**i**) Synthesis of Fluorescein modified linker: (a) AIBN/thioacetic acid/1,4-Dioxane; (b) (**i**) NaOMe, MeOH, 2 h, (**ii**) TFA: DCM (1:1), 2 h; 90%; (c) *tert*-butyl (2-aminoethyl)carbamate, EDCI, HOBt, Pyridine, 12 h, 60%; (d) TFA: DCM (2:8), 95%. (**ii**) Synthesis of Fluorescence conjugated G-AuNPs: (e) mixing of respective sugar (**1** or **2**) followed by **8** in water and methanol mixture (1:1) at RT for 12 h.

To assess the interplay between the shape and carbohydrate-mediated interactions in the zebrafish model, AuNPs were synthesized by using PEG or mannose and fluorescein linker. The required mannose-linker was synthesized as described in the literature^28^. The fluorescent-linker (**8**) was synthesized from (**3**), which was obtained by treating with thioacetic acid and azo-isobutyronitrile (AIBN), followed by one-pot de-acetylation and de-esterification to yield compound **5**. Coupling between *tert*-butyl (2-aminoethyl) carbamate and 5-Carboxyfluorescein resulted in compound **7**, which was further deprotected and *in situ* coupling of **5** gave the final fluorescein linker **8**. Fluorescent conjugated AuNPs were synthesized by mixing linker (**8**) with mannose coated AuNPs (Fig. 1). Unreacted ligands were separated by centrifugation. Similarly, pegylated hybrid AuNPs were also synthesized. The hybrid glyco-AuNPs were characterized by using UV-visible, fluorescence, (Fig. S1) zeta potential, and scanning electron microscopy (SEM) techniques. UV-visible spectra of glyco goldnanoparticles (G-AuNPs) dispalyed characteristic localised surface plasmon resonance (LSPR) peaks at 830 nm for rod-AuNPs 525 nm, for spherical-AuNPs and 723 nm, for star-AuNPs respectively. Absorbtion peak between 400–500 and emission at 524 nm, provides the evidence for conjugation of fluorescein on AuNPs. The sugar and fluorescein conjugation on AuNPs was quantified by phenol-sulfuric acid assay and UV-visible spectroscopy (Tables S1, S2 and S3).

**Figure 2:**
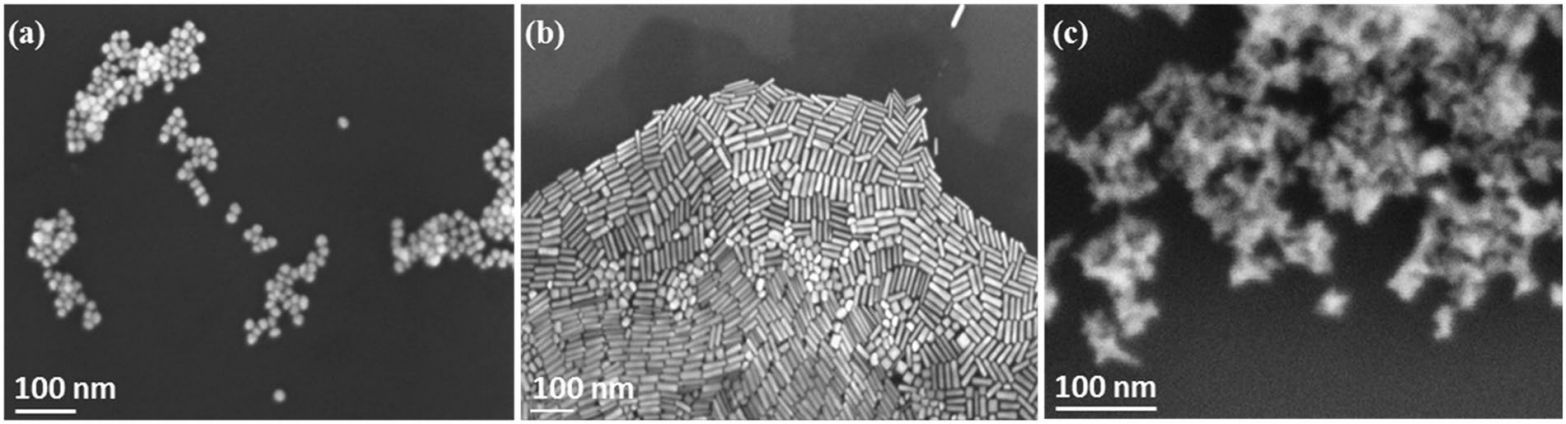
SEM images of (**a**) **S**-**2**; (**b**) **R**-**2**; (**c**) **St**-**2** glyco-gold nanoparticles.

The zeta potentials of nanoparticles were measured in both Dulbecco’s Modified Eagle’s Medium (DMEM) containing 10% fetal bovine serum (FBS) and water. The changes in zeta potentials confirmed displacement of primary surfactants by sugar and fluorescein linkers. In DMEM media, the soft layer of fetal bovine serum proteins interacts with carbohydrates moieties^29^ and alters the zeta-potential on the AnNPs further (Table 1). SEM images of mannose-AuNPs displayed almost identical size similar to native AuNPs (Fig. 2).

**Table 1:** Physical characteristics of AuNPs.

Particle	Diameter(s) (nm)	λ_max_ (nm)	ζ-potentials (mV)	
			Water	DMEM
Rod	47.6 ± 3.0 × 12.3 ± 1.5	820	30.9 ± 1.3	5.42 ± 0.6
Sphere	16.5 ± 2.0	524	−22 ± 1.2	−16.8 ± 0.3
Star	42.3 ± 2.5 × 16.1 ± 1.0	714	−27.5 ± 1.0	−6.9 ± 0.5
R-2	48.8 ± 3.5 × 12.6 ± 1.5	830	−9.5 ± 1.5	−6.1 ± 0.7
R-1	46.9 ± 3.0 × 12.0 ± 1.5	825	−28.9 ± 1.0	−2.9 ± 0.9
S-2	19.5 ± 1.5	525	−20.1 ± 1.5	−4.9 ± 0.2
S-1	19.0 ± 2.0	524	−13.7 ± 1.2	−2.1 ± 0.5
St-2	45.5 ± 1.2 × 16.5 ± 2.0	723	−21.1 ± 1.8	−3.1 ± 0.9
St-1	44.0 ± 1.0 × 16.5 ± 1.0	723	−17.1 ± 1.2	−5.2 ± 0.8

### Acute toxicity

The nanomaterial toxicity was extensively explored in the zebrafish model by exposing fishes in the nanoparticles containing tank (classical method). Although zebrafish of all life stages are utilized for toxicological studies, we chose to use adult zebrafish for this study due to their ability to absorb particles effectively. Furthermore, the nanoparticles were administration into the zebrafish *via* intraperitoneal injection instead of classical method to reduce the amount of sample consumption and toxicity can be observed with minimum number of fishes^30^. To this end, AuNPs were intraperitoneal injected to the adult zebrafish and followed their mortality. It has been observed that none of the G-AuNps showed toxicity upto 120 h, indicating that G-AuNPs are biocomatible for *in vivo* studies (Fig. S2).

### Biodistribution and sequestration studies of G-AuNPs in Zebrafish model

**Figure 3:**
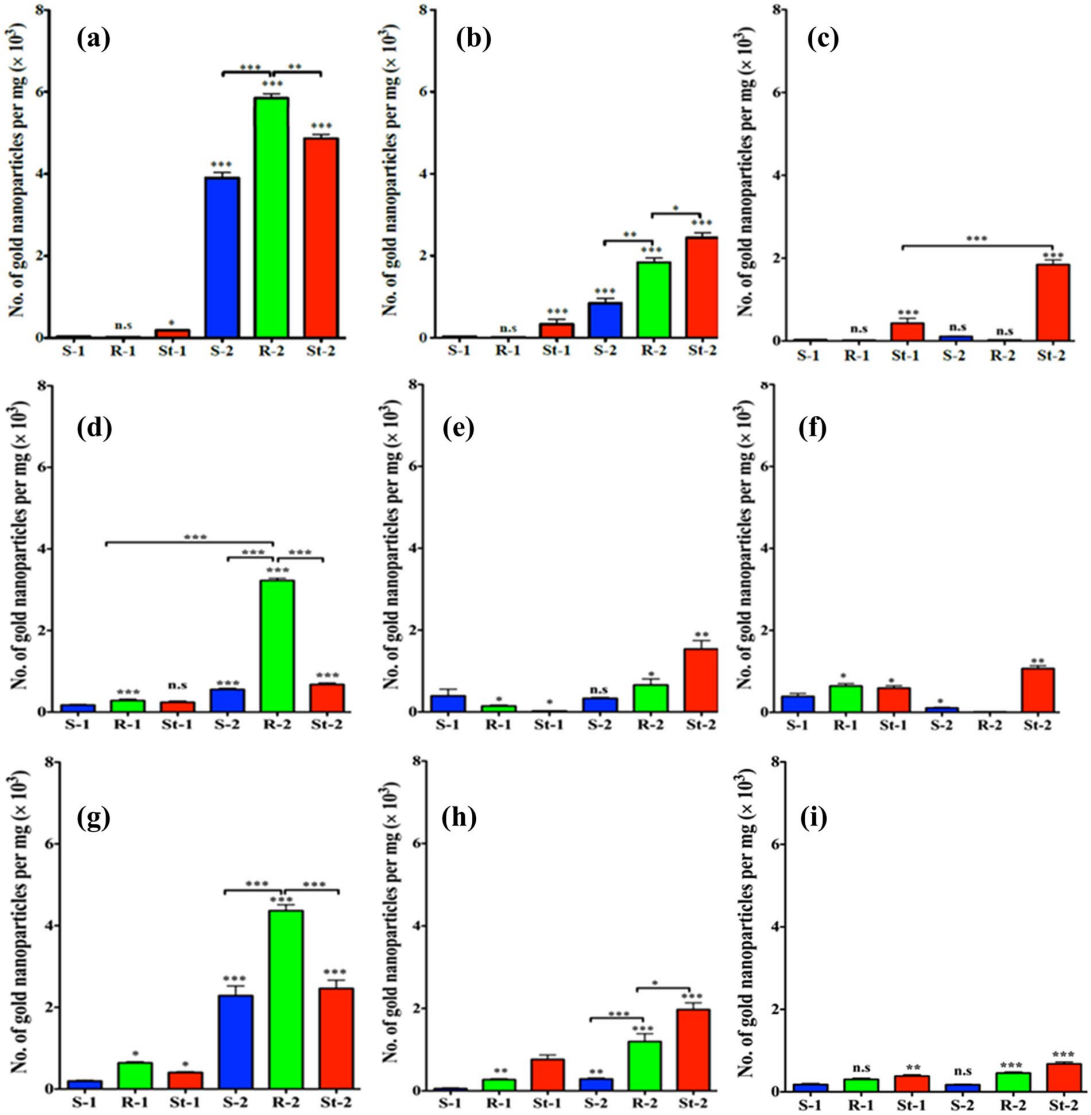
Statistical analysis of ICP-MS data of digestive system, heart and swimming bladder of zebrafish at different time intervals: (**a**) digestive system −4 h; (**b**) digestive systm −24 h; (**c**) digestive system −48 h; (**d**) heart −4 h; (**e**) heart −24 h; (**f**) heart −48 h; (**g**) swim bladder −4 h; (**h**) swim bladder −24 h; (**i**) swim bladder −48 h. Data are presented as mean ± SEM for three independent experiments (***P < 0.001, **P < 0.01 *P < 0.05 and n.s = not significant).

Since the AuNPs were non-toxic, it is worth to study their biodistribution and sequestration to target the specific organ using different shapes^31–33^ and carbohydrate receptors. To this end, PEGylated and mannose-AuNPs (5 µg/g) of different shapes were intraperitoneally injected into zebrafish (Fig. S3). After 4 h, 24 h and 48 h exposure, fishes were sacrificed and different organs (brain, eye, heart, muscles, swim bladder and digestive system) were dissected. We examined the biodistribution of AuNPs into the various organs by quantifying the gold concentration using inductively coupled plasma mass spectrometry (ICP-MS). Figure 3 shows the organ-specific AuNPs sequestration at different time intervals. Our results demonstrated remarkable sensitivity of the zebrafish towards mannose-AuNPs compared to PEG-AuNPs. Mannose-AuNPs were sequestered in the digestive system, swim bladder and heart, but not in the brain, muscles and the eyes (Fig. S4). However, the intrinsic shape of the AuNPs generated unexpected interrelationship on the number of AuNPs uptake and clearance. As illustrated in Fig. 2(a), the digestive system exhibited maximum mannose AuNPs sequestration after 4 h. However, after 24 h and 48 h a drastic difference in the shape dependent accumulation of nanoparticles was observed. Elongated particles (**R**-**2**) accumulated in higher number in the initial state and got cleared after 48 h, whereas, the **St**-**2** was accumulated in steady state and sequestered for extended periods of time as compared to **S**-**2**. PEGylated-AuNPs had the least sequestration, demonstrating the inter-relationship between shapes and carbohydrate-mediated interactions (Fig. 3). The sequestration of mannose-AuNPs further support the presence of sugar receptors, either in the form of dendritic cell-specific intercellular adhesion molecule-3-grabbing non integrin (DC-SIGN) or other C-type lectins. Similar trends were observed in heart, indicating the broad distribution of mannose receptors in the zebrafish model. In the swim bladder, accumulation was higher for mannose nano-rod (**R**-**2**) after 4 h^34, 35^. However, after 24 h and 48 h, clearance of **R**-**2** and slow sequestration of star- nanoparticles further confirm the therapeutical value of star-AuNPs.

The observed differences in the rate of biodistribution and sequestration of different shapes of AuNPs could be attributed to several physical factors associated with the nanoparticles and the chemical compositions as well. The physical factors such as aspect ratio of rod shape nanoparticles induced fast uptake and clearance from the system compared to spherical counter parts. While friction coefficient of nanoparticles (NPs), which is high in the star-shape AuNPs^36^ and tumbling motion of NPs underflow invivo system is expected to be influence the sequestration of the star AuNPs. In addition, mannose based interactions could also influence the uptake mechanism. However, these interactions is more precise with higher glycans conjugated AuNPs. Finally, clearance of AuNPs was quantified by gold concentration in the water tank. As illustrated in Fig. S5, the PEG-AuNPs clearance from the zebrafish system much faster than the mannose-AuNPs counterparts. Further, star-AuNPs slow clearance further illustrates the significance of shapes in the *in vivo* studies.

**Figure 4:**
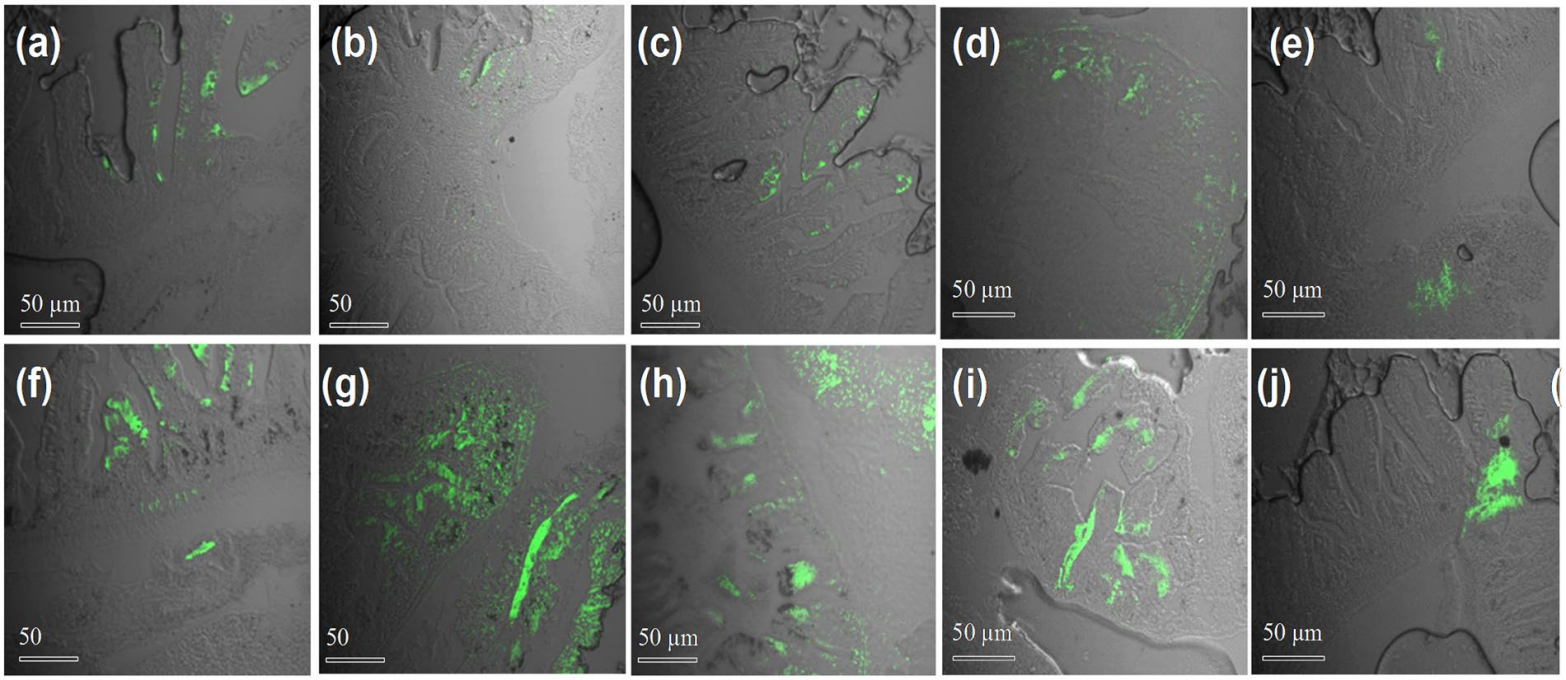
Confocal images of zebrafish digestive system injected with Fluorescein conjugated G-AuNPs after 4 h: (**a**) S-1, 4 h; (**b**) R-1, 4 h; (**c**) St-1, 4 h; (**d**) R-2, 24 h; (**e**) R-2, 48 h; (**f**) S-2, 4 h; (**g**) R-2, 4 h; (**h**) St-2, 4 h; (**i**) St-2, 24 h; (**j**) St-2, 48 h.

The digestive system accumulation was qualitatively analyzed by confocal imaging of the tissue sections^37^. Figure 4 explicitly describes the relative fluorescent intensity of fluorescein conjugated G-AuNPs after 4 h. As expected, PEGylated nanoparticles were least sequestered in the digestive systems. While, mannose-AuNPs seem to taken up substantial by the digestive system. The time dependent, confocal imaging of **St**-**2** clearly showed that St-mannose-AuNPs sequestered in the digestive system for a long period (Fig. 4) compared to **R**-**2**. overall, the shape and mannose conjugation on the nanoparticles showed marked differences in the biodistribution and sequestration.

## Conclusion

Here, we describe successfully constructed bifunctional fluorescent glyco-gold-nanoparticles to probe its *in vivo* efficiency using zebrafish model. The intraperitonial injection of these nanoparticles into the adult zebrafish resulted very low toxicity, indicating the potential use of these nanoparticles for drug delivery and imaging studies. Using ICP-MS analysis and confocal imaging, we have demonstrated that the rate of bio-distribution of the AuNPs varies with its shape and mannose conjugation. Rod-AuNPs showed faster uptake and clearance. In contrast, star-AuNPs showed slow and long sequestration compared to other two shapes. Moreover, AuNPs showed remarkable sensitivity towards mannose compared to PEG linker.

## Methods

### Synthesis of glyco-gold nanoparticles

Gold nanoparticles of different shapes were synthesized by applying literature procedures^38–40^. 0.5 ml of 1 mM thiol PEG linker (**1**) /thiol mannose linker (**2**) and purified by centrifugation, followed by mixed with 0.1 mM of **8** with 0.5 ml of the AuNPs solution. After incubation at room temperature for 12 h, the modified Au nanostructures were separated by centrifugation and re-dispersed in deionized water.

### Zeta potential studies

We used a zeta potential analyzer to measure the surface potential of AuNPs. In the measurement, we applied unit field strength (1 Volt per meter) to the AuNP solution. We measured zeta potential of different shapes of AuNPs in water. In the case of DMEM medium, we incubated all AuNps in DMEM medium (containing 10% FBS) for 24 h and purified by centrifugation and measured zeta potential.

### Phenol-sulfuric acid method to quantify sugars on AuNPs

The concentration of mannose sugars on AuNPs were determined by the phenol-sulfuric acid method. 50 μL sugar functionalized-AuNPs were added to concentrated sulfuric acid (130 μL, 100%) and aqueous phenol solution (5% w/v, 30 μL) in the test tube and heated to 90 °C. After 5 min, the solution was cooled to room temperature and the absorbance coefficient at 490 nm was measured. AuNPs as such in sulfuric acid was used as a control. The sugar concentration was estimated by comparing the absorption of the sample with a standard curve. The compound **8** concentration was measured by using absorption coefficient of 4-carboxy fluorescein.

### Zebrafish model

Local wild-type zebrafish strain weighing approximately 500–600 mg (2–3 months old) were maintained under standard laboratory conditions at 28 °C under 14:10 h light/dark cycle conductivity of 350 μS of the water maintained at pH 7.2–7.4. The surgical procedures were performed in accordance with Institutional Animal Ethical Committee regulation, set up by CPCSEA, Govt. of India.

### Acute toxicity determination

Zebrafishes were anesthetized with 2-phenoxyethanol and -AuNPs were injected. The number of zebrafish in each experimental and control group was 6 in each group.

### Sample preparation for ICP-MS analysis

Zebrafish were anesthetized with 2-phenoxyethanol and injected 2 µl contain 5 µg/g nanoparticles of rod, sphere and star shape AuNPs via intraperitoneally using *catheter* implantation tubing attached to a cut 22-G needle tip at one end and another end attached to Hamilton syringe. After 4 h, 24 h and 48 h injection, fishes were sacrificed and organs were collected. Organs were homogenized with 400 μl of *aqua regia* at 95 °C for 4 h. All digested samples were centrifuged at 5000 rpm for 10 min to remove debris. Then each digested samples were diluted to 6 ml with Millipore water. The concentration of Au, determined by ICP-MS (Thermo-Fisher Scientific, Germany), was converted into the number of AuNPs per one mg per gram of the organ.

### Confocal imaging studies

Zebrafish were anesthetized with 2-phenoxyethanol and injected with 2 µl contain 5 µg/g nanoparticles of FITC conjugated G-AuNPs *via* intraperitoneal injection. After 4 h, 24 h and 48 h of injection, fishes were sacrificed and digestive system was collected. Organ was fixed with 4% paraformaldehyde and 4% glutaraldehyde followed by dehydration with the gradient increase of ethanol (75%, 95%, 100%) for 30 min each, further with xylene for 1 h and then fixed in paraplast. Blocks were stored in −10 °C for 12 h before proceeding to section. 10 µm thicknesses of sections were cut by using *Leica* microtome instrument and sections were collected on PLL-coated glass plates. The sections were washed with xylene to remove excess of paraplast. After drying, sections were fixed with mounting media. Sequestration of AuNPs in digestive system was analyzed by confocal fluorescence microscopy using CLSM (Zeiss LSM 710) microscope. The excitation wavelength was 450 nm, detection wavelength was 510 nm and 25 X objective was used to image digestive system sections.

### Statistical analysis

Statistical comparisons were done using the Student *t* test or one-way ANOVA. The p < 0.05 is considered to be statistical significance.

## Electronic supplementary material

Mapping the Glyco-Gold Nanoparticles of Different Shapes Toxicity, Biodistribution and Sequestration in Adult Zebrafish
