# Supplementary material for: Mapping the Glyco-Gold Nanoparticles of Different Shapes Toxicity, Biodistribution and Sequestration in Adult Zebrafish
Source: Sci Rep. 2017 Jun 26;7:4239. doi: 10.1038/s41598-017-03350-3 (PMC5484690; doi:10.1038/s41598-017-03350-3)
Supplement: Supplementary file 1 — Mapping the Glyco-Gold Nanoparticles of Different Shapes Toxicity, Biodistribution and Sequestration in Adult Zebrafish [file 41598_2017_3350_MOESM1_ESM.doc]

Supporting Information

Mapping the Glyco-Gold Nanoparticles of Different Shapes Toxicity, Biodistribution and Sequestration in Adult Zebrafish

Sivakoti Sangabathuni,^[a]^ Raghavendra Vasudeva Murthy,^[b]^ Preeti Madhukar Chaudhary,^[a]^ Balamurugan Subramani,^[a]^ Suraj Toraskar,^[a]^ and Raghavendra Kikkeri*^[a]^.

^a^Indian Institute of Science Education and Research, Dr. Homi Bhabha Road, Pune-411008, India.

^b^Department of Clinical and Experimental Medicine, Linkoping University, Sweden.

1. **Materials and Methods**
   1. **General Instructions.** All chemicals were reagent grade and used as supplied except where noted. Analytical thin layer chromatography (TLC) was performed on Merck silica gel 60 F254 plates (0.25 mmol). Compounds were visualized by UV irradiation or dipping the plate in CAM/ninhydrin solution followed by heating. Column chromatography was carried out using force flow of the indicated solvent on Fluka Kieselgel 60 (230–400 mesh). ^1^H and ^13^C NMR spectra were recorded on Jeol 400 MHz, using residual solvents signals as an internal reference (DMSO d_6_ δ_H_, 2.5, 3.3 ppm, δ_C_ 39.5 ppm and CD_3_OD δ_H_ 3.31 ppm, δ_C_ 49.0 ppm). The chemical shifts (δ) are reported in ppm and coupling constants (*J*) in Hz. UV-visible measurements were performed with Evolution 300 UV-visible spectrophotometer (Thermo Fisher Scientific, USA). Fluorescence spectra were recorded in FluoroMax-4 spectrofluorimeter (Horiba Scientific, U.S.A.).
2. **Synthesis of FITC conjugated start G-AuNPs.**

**Synthesis of Compound (7):** 5-carboxyl-fluorescein **6** (0.2 g, 0.72 mmol), HOBt (0.08 g, 0.53 mmol) and EDCI (0.153 g, 0.79 mmol) were stirred in dry pyridine at ice cold condition for 15 min. To this solution *tert*-butyl (2-aminoethyl)carbamate (0.127 g, 0.79 mmol), was slowly added. The corresponding reaction mixture was allowed to stir for 12 h. After completion of reaction (monitored by TLC), the crude reaction mixture was concentrated and purified by column chromatography with MeOH : DCM (20 : 80) to afford compound **7**. Yield: 0.2 g, (72 %). ^1^H NMR (400 MHz, DMSO-d^6^) δ 10.24 (s, 2H), 8.84-8.68 (m, 1H), 8.25 – 8.07 (m, 1H), 8.02 – 7.96 (m, 1H), 7.72 (d, *J* = 8.4 Hz, 1H), 7.54 (t, *J* = 7.6 Hz, 1H), 7.43-7.37 (m, 1H), 6.98-6.86 (m, 1H), 6.70 (s, 1H), 6.62-6.53 (m, 3H), 3.22 (q, 1H), 3.18-3.13 (m, 2H), 3.05 (q, *J* = 6.2 Hz, 1H), 1.38 (s, 9H). ^13^C NMR (100 MHz, DMSO-d^6^) δ 168.67, 168.52, 165.28, 162.77, 160.08, 156.20, 152.30, 129.70, 129.54, 128.27, 127.68, 126.86, 124.91, 123.82, 119.58, 113.21, 113.14, 110.10, 109.62, 109.58, 102.78, 102.72, 78.16, 78.08, 49.07, 36.25, 28.70, 28.63. HRMS for C_28_H_26_N_2_O_8_ (M+H)^+^ calculated m/z = 519.1767; Found, 519.1767.

**Synthesis of Compound (8):** Compound **7** (0.2 g, 0.38 mmol) was dissolved in 1 ml of TFA : DCM ( 1 : 1) and allowed to stir for 2 h. After completion of the reaction, the crude mixture was concentrated and directly taken into next step, to this HOBt (0.06 g, 0.38 mmol), EDCI (0.11g, 0.57 mmol) and compound **5** (0.19 g, 0.57 mmol) were added in pyridine. The corresponding reaction mixture was allowed to stir for 12 h. After completion of reaction (monitored by TLC), the crude reaction mixture was concentrated and purified by column chromatography with MeOH : DCM (20 : 80) to afford compound **8**. Yield: 0.08 g (41 %). ^1^H NMR (400 MHz, CD_3_OD) δ 7.89 (d, *J* = 8.3 Hz, 2H), 7.75 (d, *J* = 8.3 Hz, 2H), 7.58 – 7.48 (m, 5H), 3.75 (t, *J* = 6.3 Hz, 2H), 3.68 – 3.57 (m, 12H), 3.47 (t, *J* = 6.6 Hz, 2H), 2.69 (t, *J* = 7.2 Hz, 2H), 2.57 (t, *J* = 6.2 Hz, 2H), 1.70 – 1.66 (m, 2H), 1.57 (t, *J* = 6.9 Hz, 2H), 1.52 – 1.42 (m, 2H), 1.41 – 1.26 (m, 16H). ^13^C NMR (100 MHz, CD_3_OD) δ 173.90, 141.33, 128.19, 127.01, 126.97, 125.86, 117.22, 110.05, 70.98, 70.17, 70.16, 70.08, 70.01, 69.75, 66.42, 38.44, 34.43, 34.35, 29.33, 29.25, 29.22, 29.19, 28.91, 28.82, 28.05, 26.23, 25.82. HRMS for C_43_H_56_N_2_O_11_S (M+Na)^+^ calculated m/z = 831.9738; Found, 832.5280.

1. **Uv-visible and physical characteristic of the complexes.**

**(a) (b)**

**(c) (d)**

**(e) (f)**

**Figure S1**. UV-visible spectra of FITC conjugated sphere, rod and star shaped AuNPs in water (**a, c, e)** and Fluorescene spectra of sphere, rod and star (**b, d, f**).

**Table S1.** Quantification of sugar concentration on FITC conjugated sphere, rod and star shape G-AuNPs.

| **S.No** | **Nanoparticles** | **Concentration(mg/mL)** |
| --- | --- | --- |
| **1** | Rod-Man | 1.7 ± 0.12 |
| **2** | Sphere-Man | 1.9 ± 0.3 |
| **3** | Star-Man | 1.7 ± 0.9 |

**Table S2.** Quantification of Fluorescein after conjugation with 4-carboxy fluorescein on sphere, rod and star shape G-AuNPs.

| **S.No** | **Nanoparticles** | **FITC Concentration(µg/mL)** |
| --- | --- | --- |
| **1**  **2**  **3**  **4**  **5**  **6** | S-1  S-2  R-1  R-2  St-1  St-2 | 25.54 ± 3.4  36.12 ± 2.74  41.4 ± 3.41  45.5 ± 4.78  29.23 ± 1.54  38.01 ± 2.45 |

**Table S3.** Quantification of quantum yield of fluorescein conjugated nanoparticles

| **Peptide** | 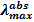 | ***A*_max_** | 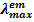 | **φ** |
| --- | --- | --- | --- | --- |
| **S-1**  λ*^em.^*[nm]  *_max_* | 475 | 0.10184 | 515 | 0.23 |
| **R-1** | 475 | 0.9878 | 515 | 0.25 |
| **St-1** | 475 | 0.9925 | 515 | 0.27 |

Quantum yield of 5-carboxy fluorescein is 0.30^2^

**Figure S2.** Percentage of motality of zebrafish with respect to concentration in presence of Glyco-AuNPs at 120 h.


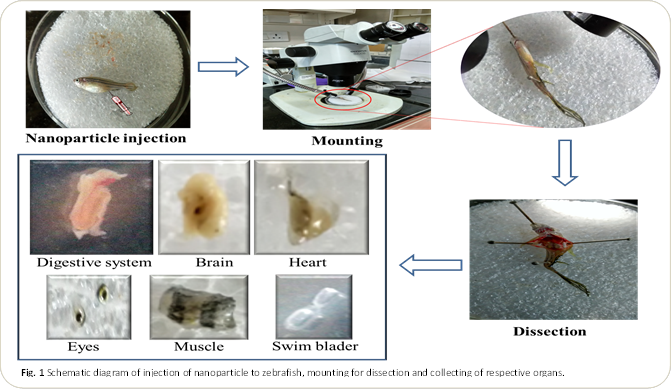


**Figure S3.** Schematic diagram of injection of G-AuNPs into zebrafish, mounting for dissection and collection of respective organs.


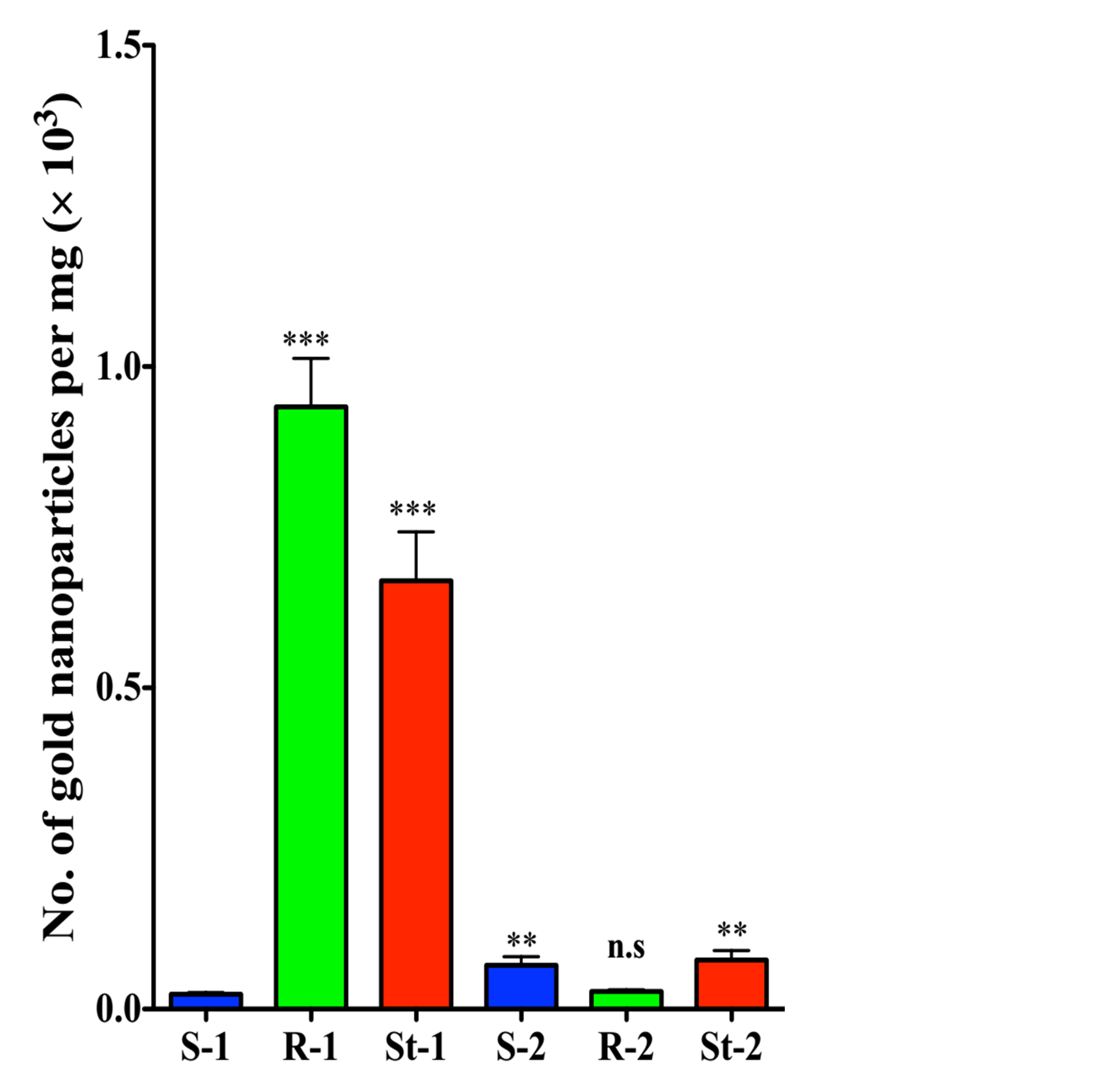

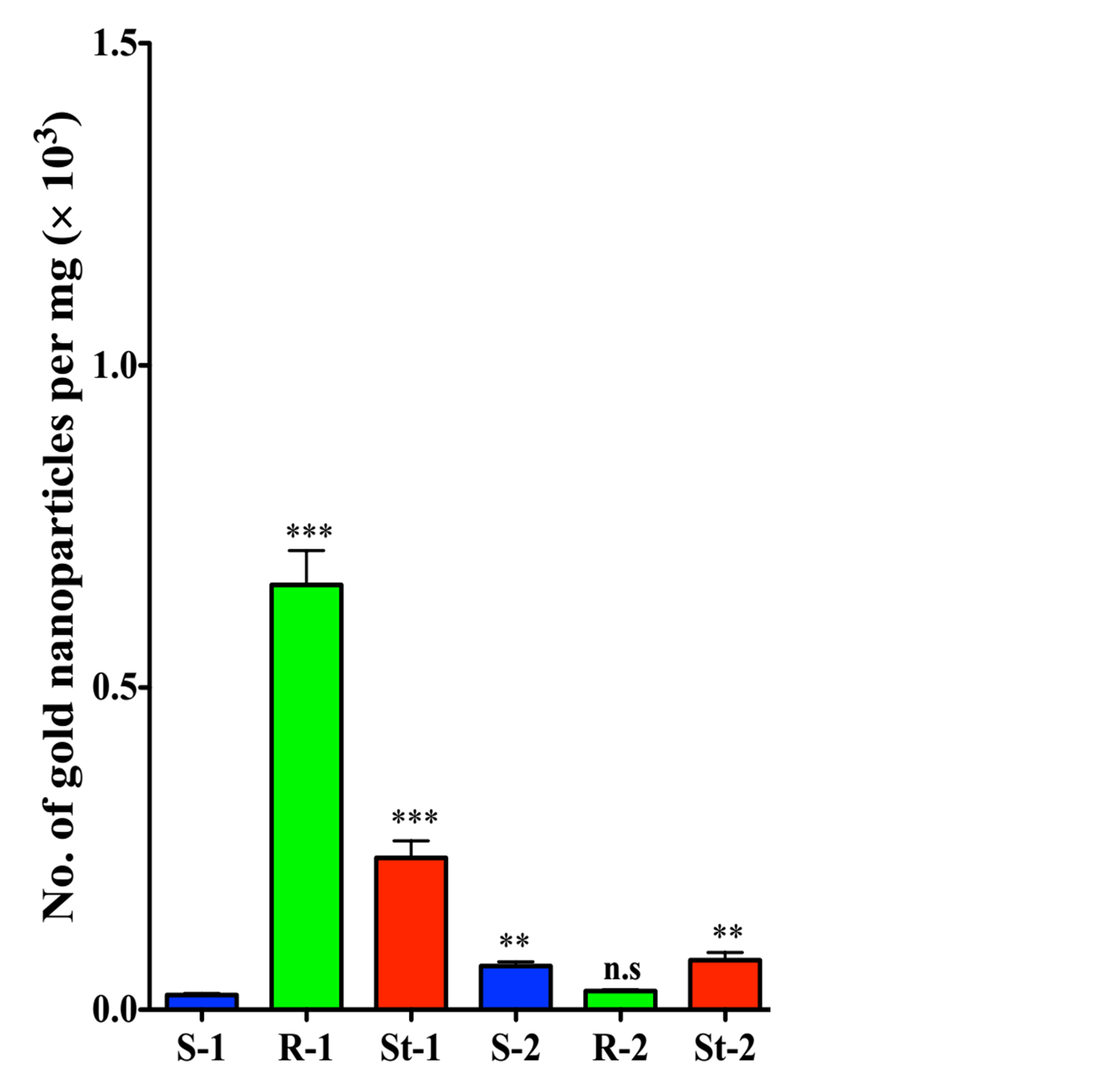

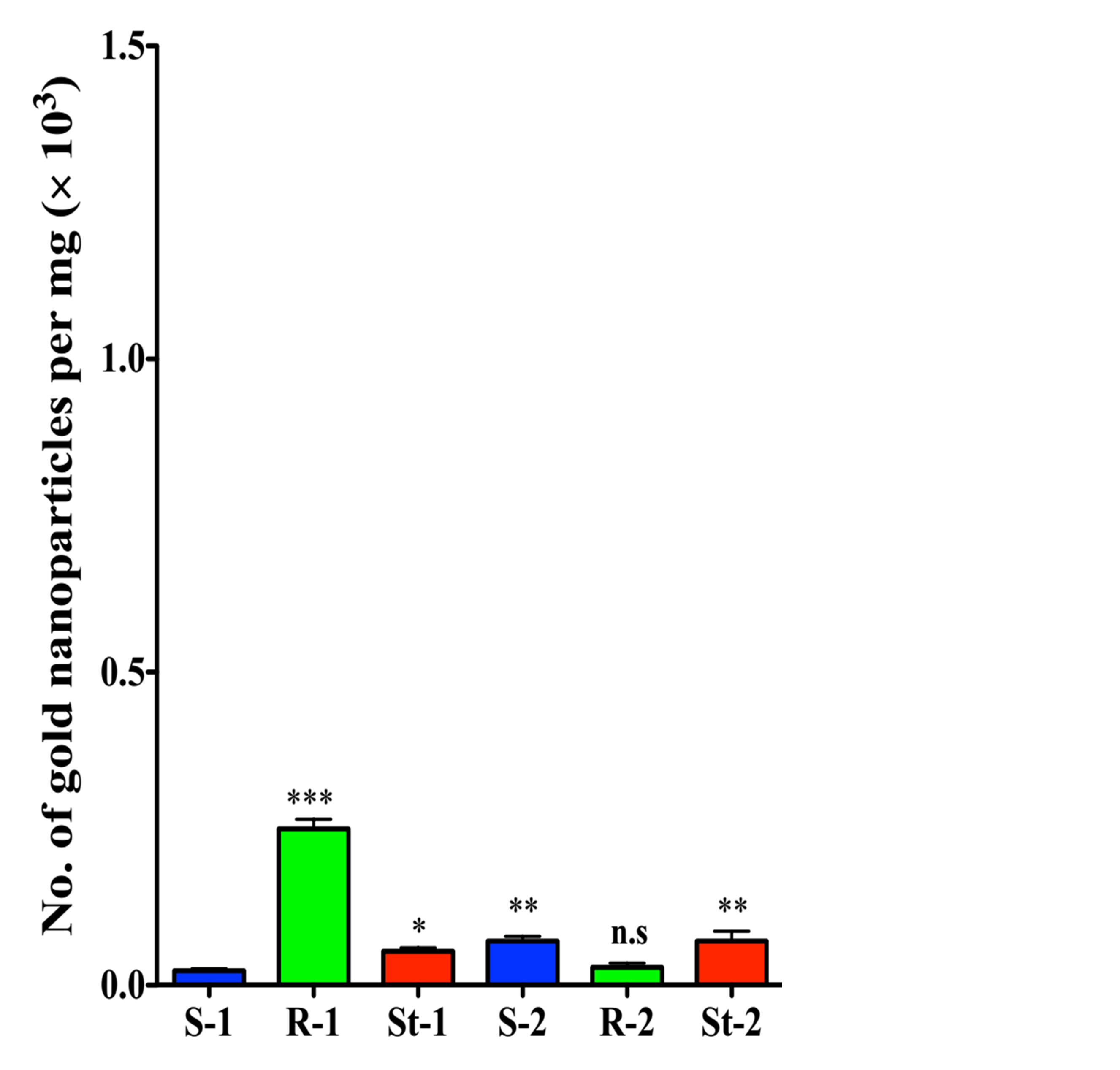

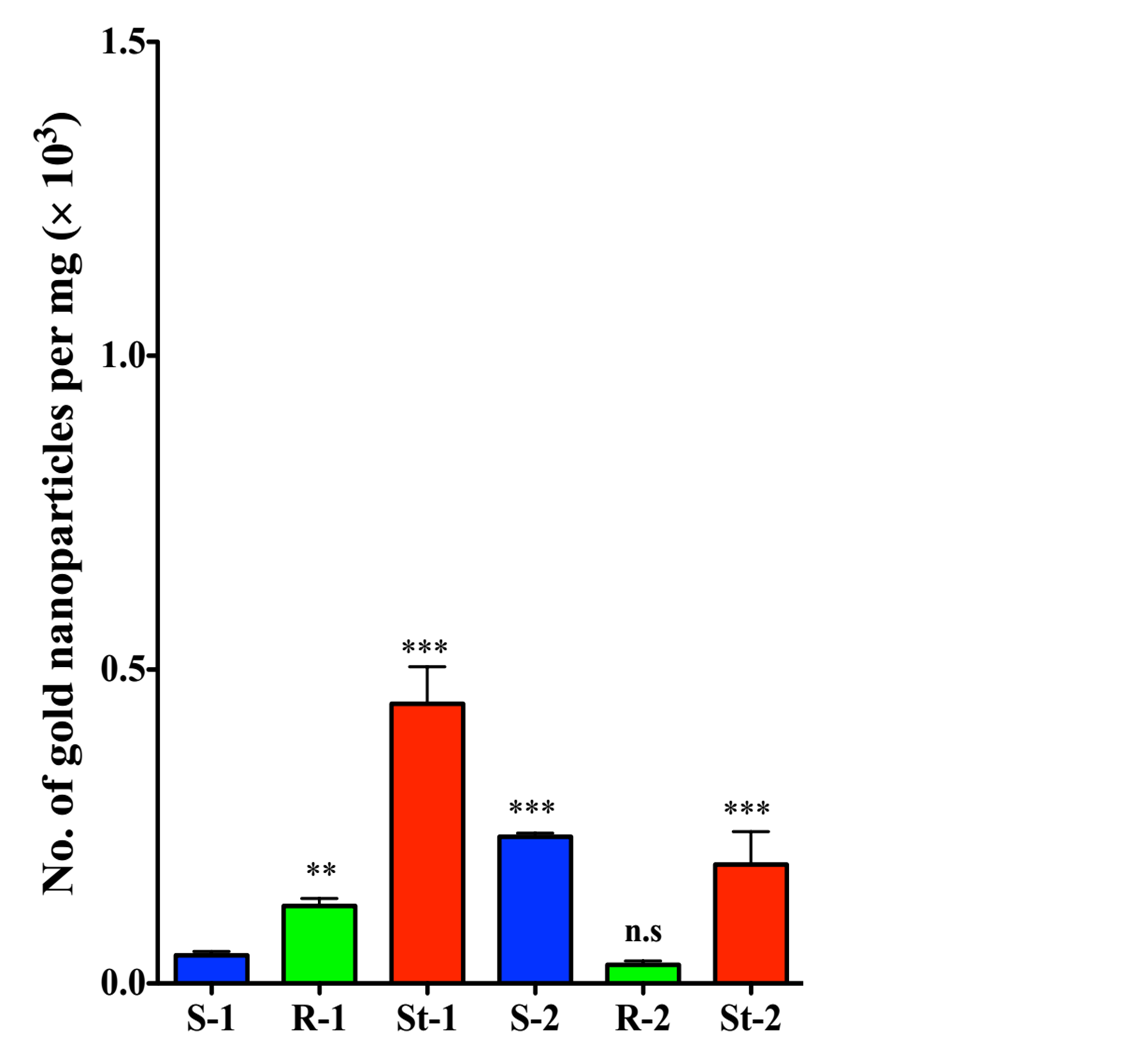

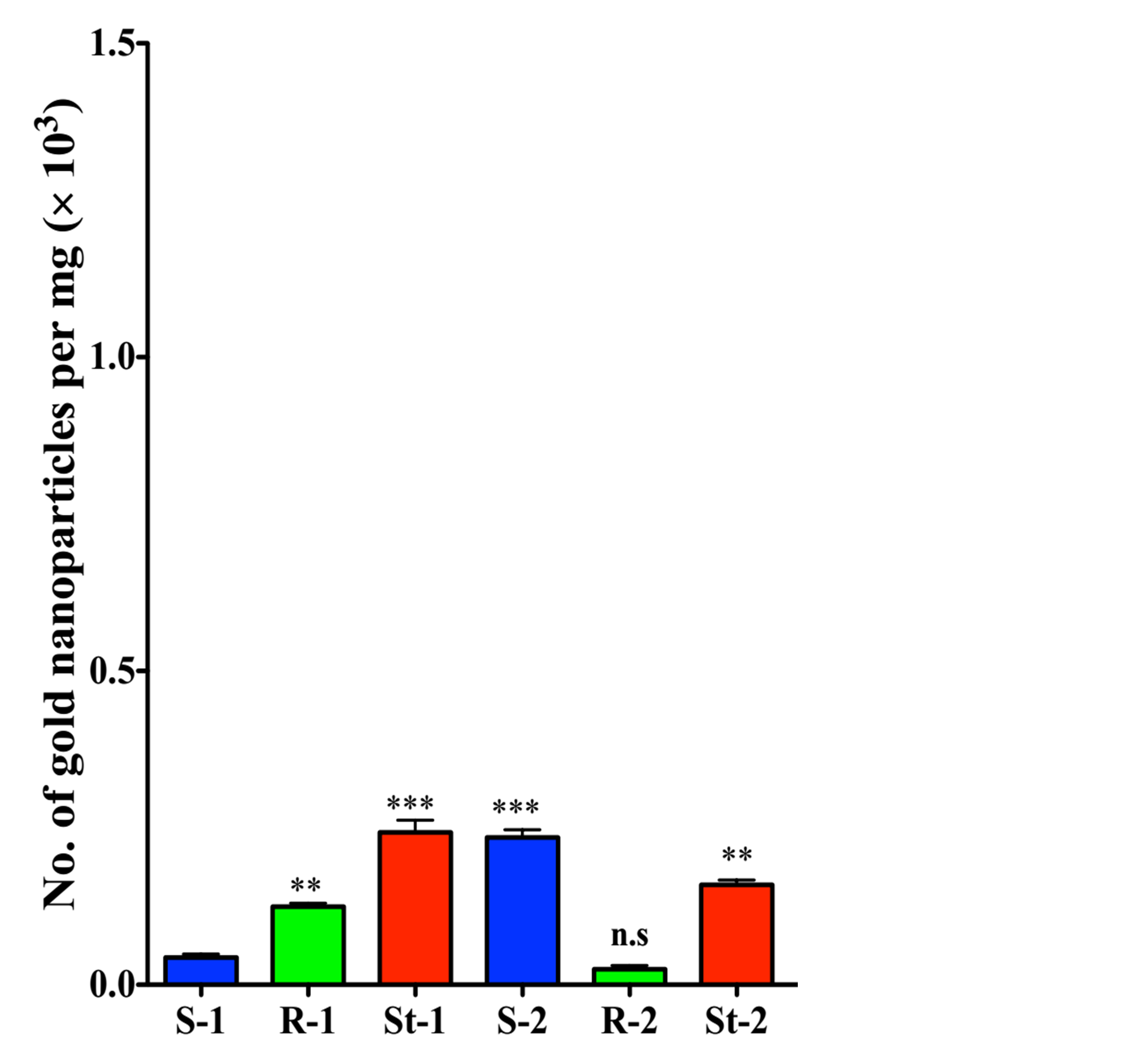

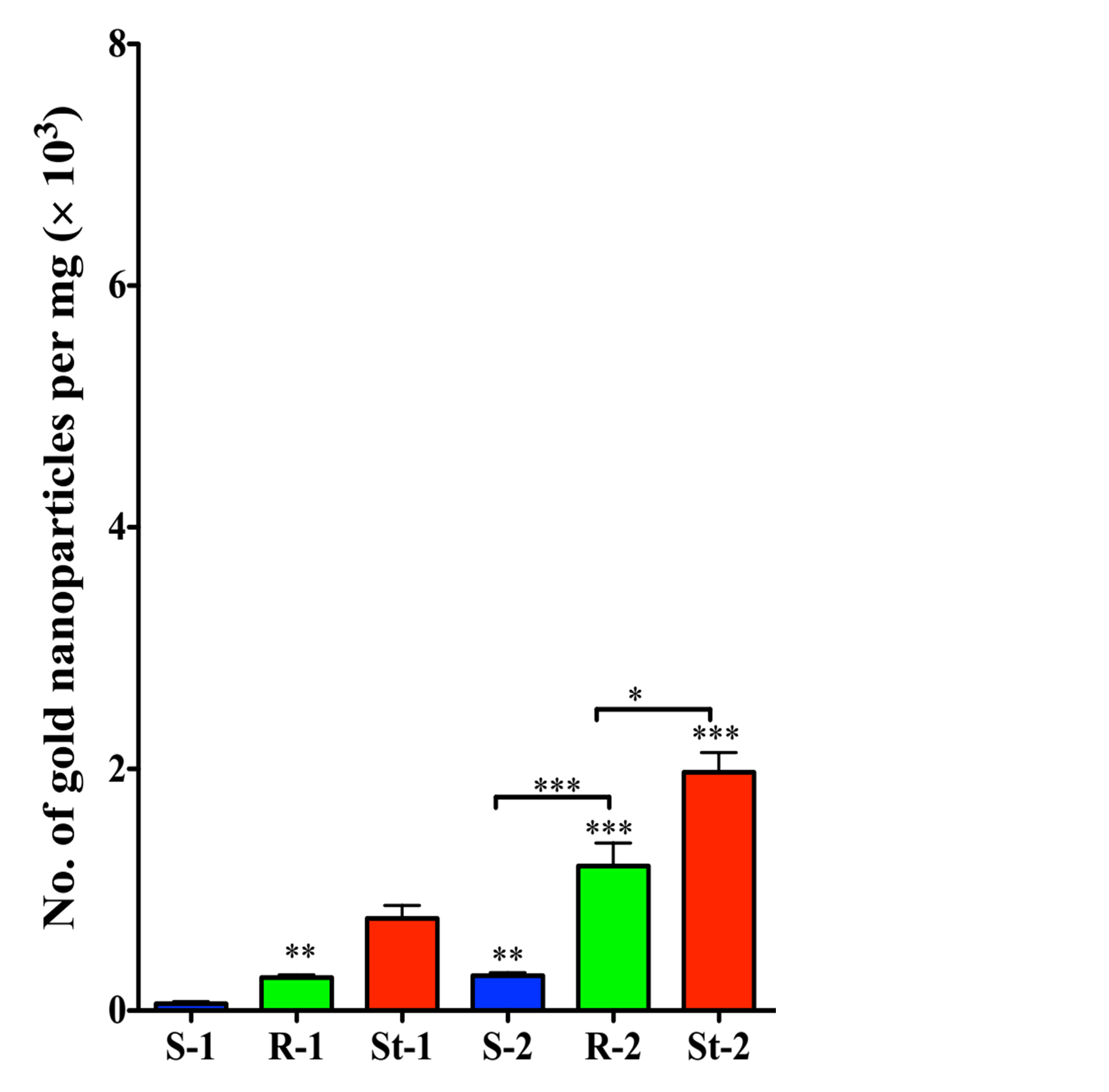

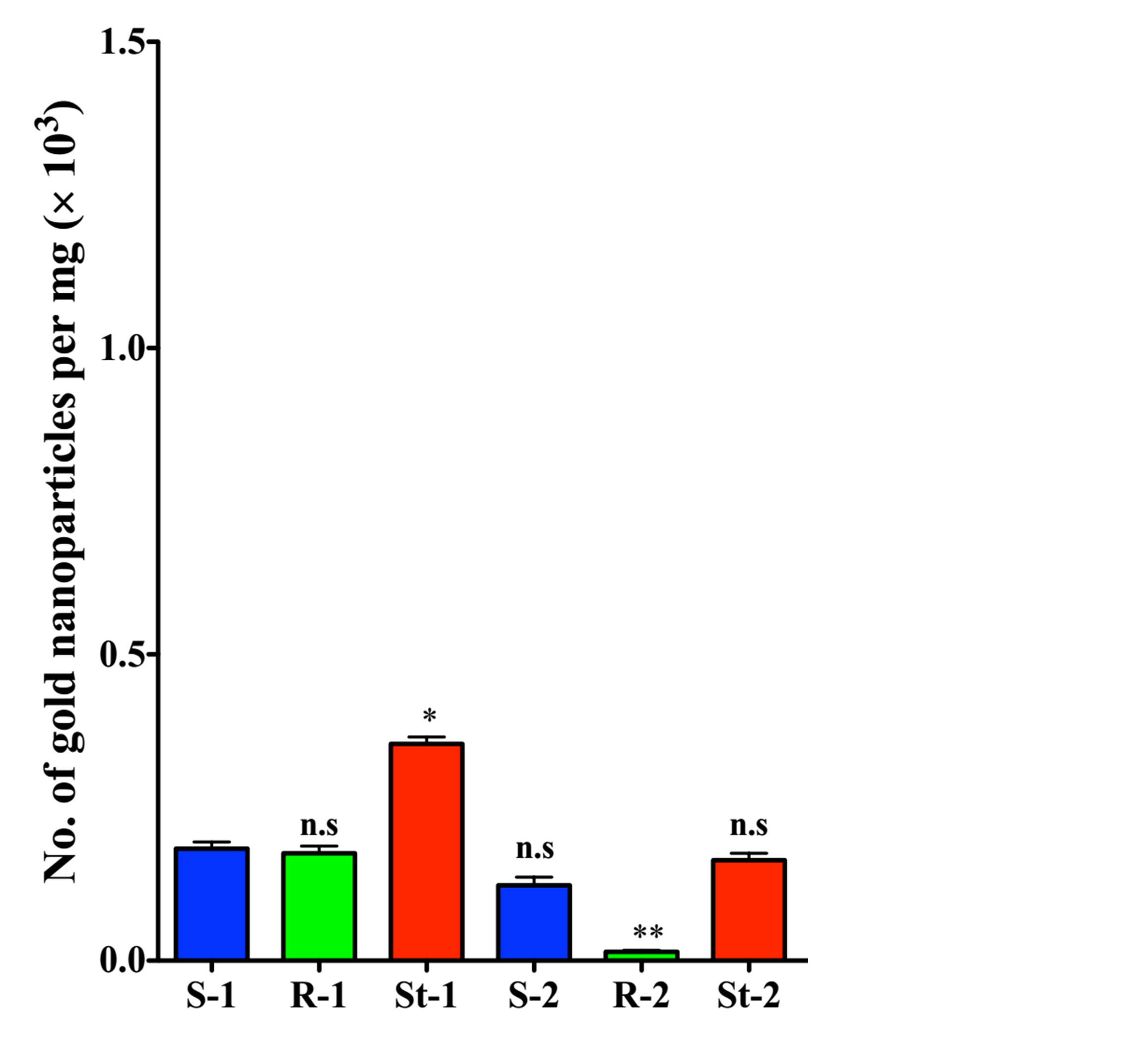

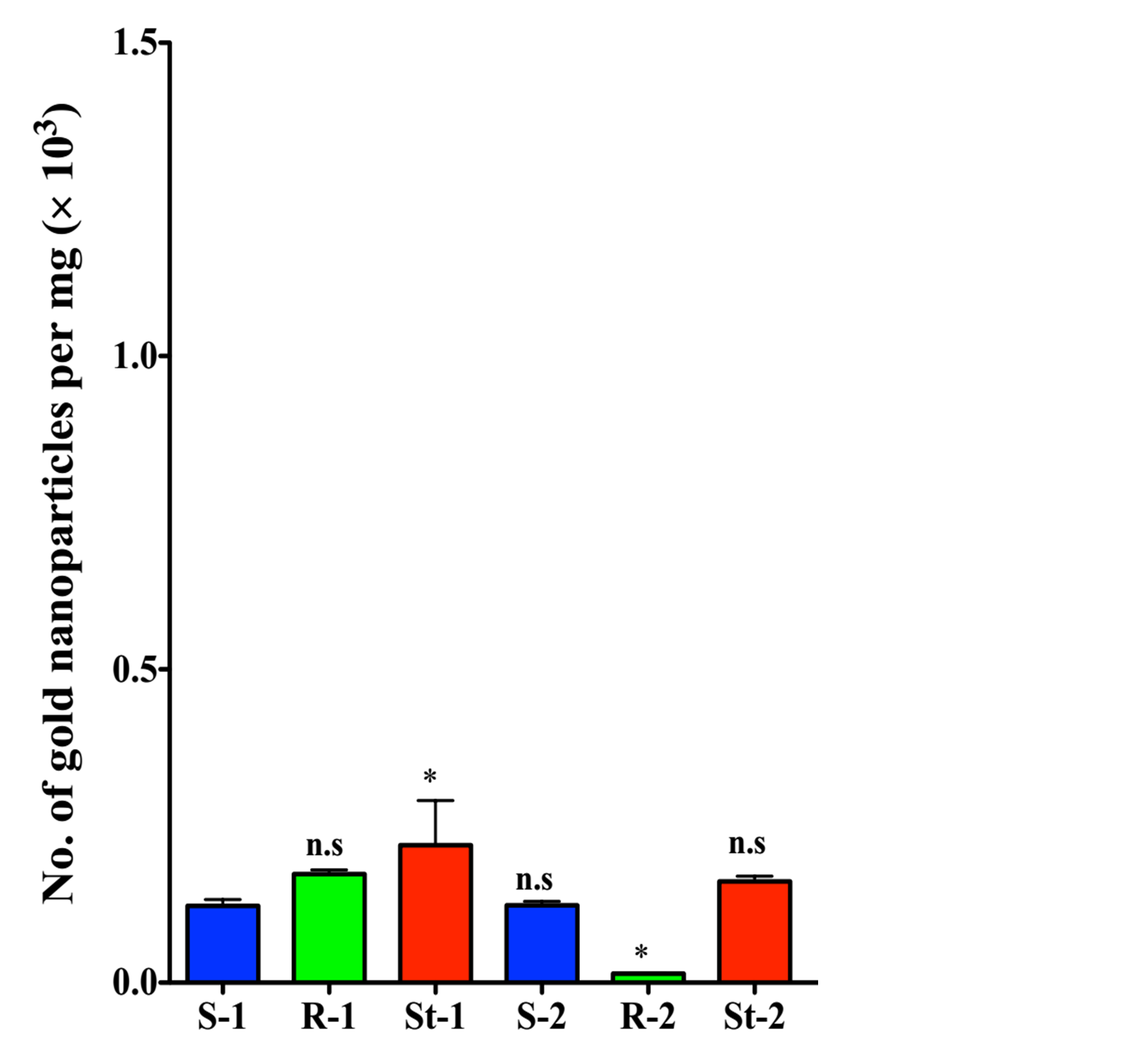

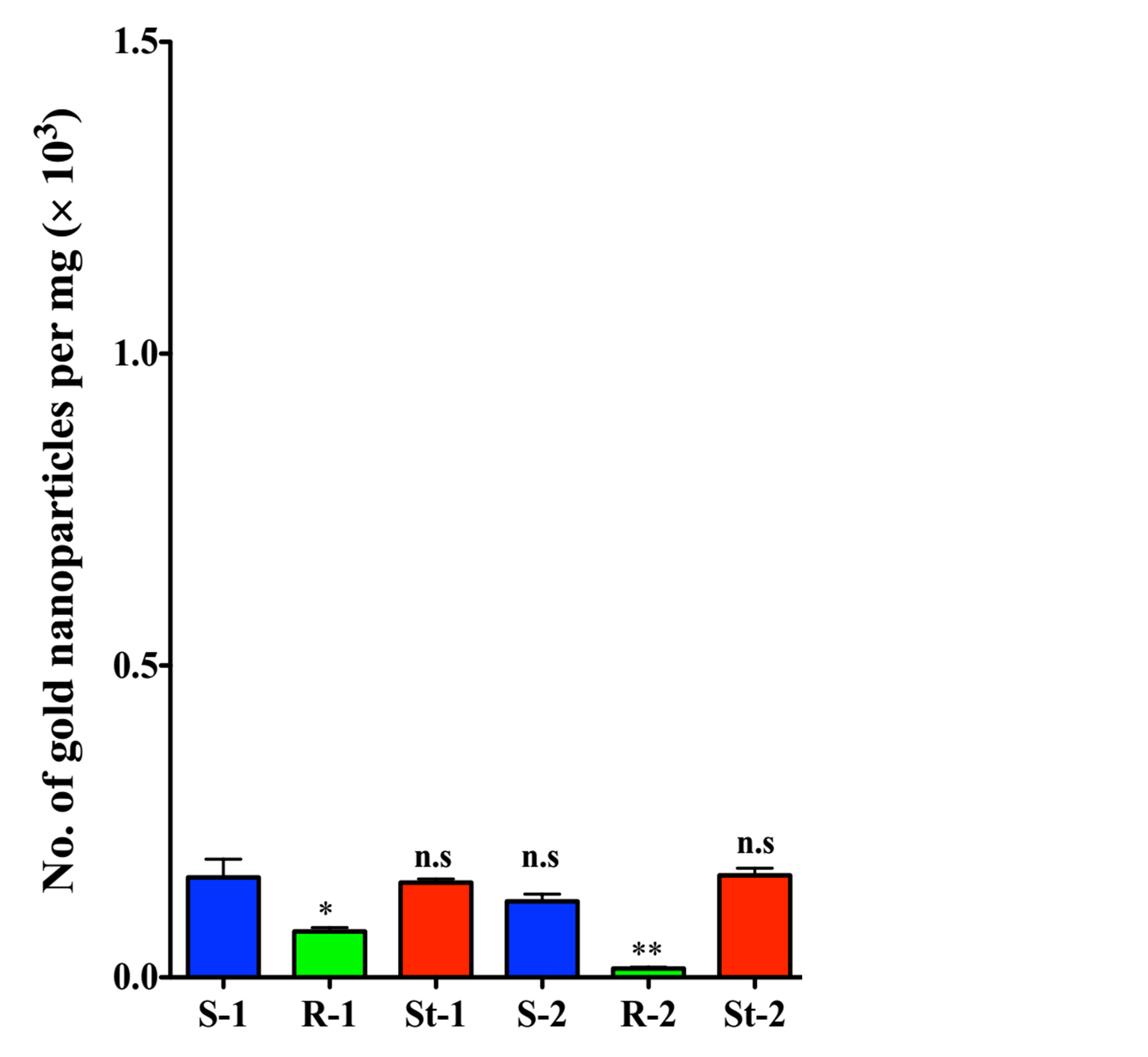


1. **(b) (c)**

**(d) (e) (f)**

**(g) (h) (i)**

**Figure S4.** Statistical analysis of ICP-MS data of brain, eye and muscles of zebrafish at different time intervals: (a) brain -4h; (b) brain -24 h; (c) brain-48 h; (d) eye -4 h; (e) eye-24 h; (f) eye-48 h; (g) muscles -4 h; (h) muscles-24 h; (i) muscles-48h. (***P<0.001, **P<0.01 *P<0.05 and n.s = not significant).

**(a) (b) (c)**

**Figure S5.** ICP-MS data of Clearance of Glyco-goldnanoparticles after (a) 4 h; (b) 24 h; (c) 48 h. administration into zebrafish. (***P<0.001, and n.s = not significant).

1. **NMR Spectras of FITC conjugated linker**

**(d) (e) (f)**

**(g) (h) (i)**


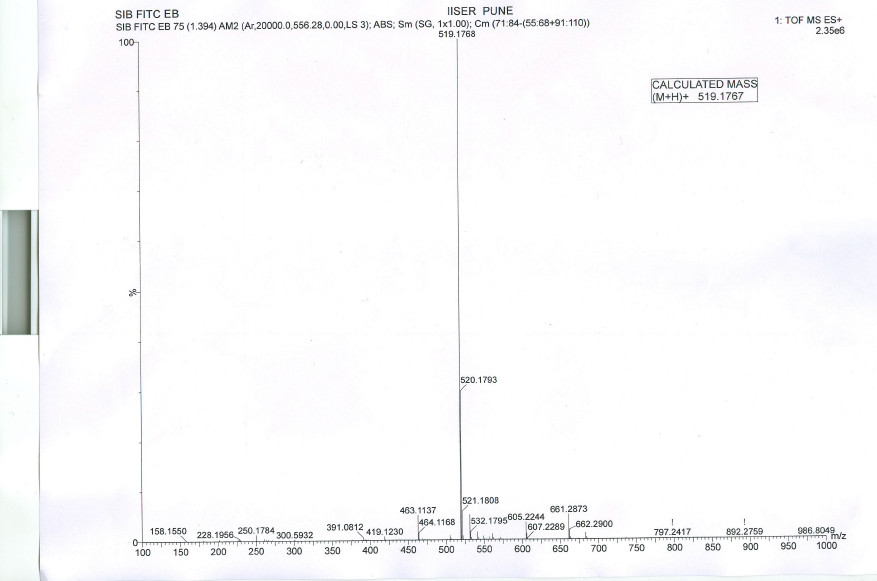


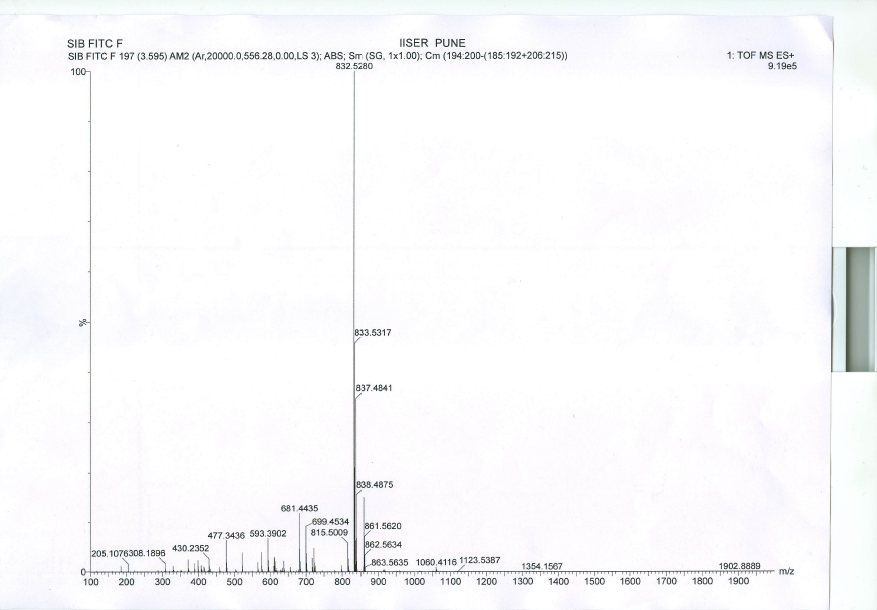


1. References
2. P. M. Chaudhary, S. Sangabathuni, R. V. Murthy, A. Paul, H. V. Thulasiram and R. Kikkeri, *Chem. Commun*., 2015, **51,** 15669.
3. S. Massou, R. Albigot, M. Prats, *Biochemical Education*, 2000, **28**, 171.
